# Health Systems Science Scholars Program: National Faculty Development Program

**DOI:** 10.1007/s40670-026-02819-z

**Published:** 2026-07-13

**Authors:** Sally A. Santen, Jed D. Gonzalo, Resney Gugwor, Judee Richardson, Jung G. Kim, Stephanie R. Starr, Luan Lawson, Robin R. Hemphill, Timothy J. Reeder, Rosalyn Maben-Feaster, Kevin Heckman, Maya M. Hammoud

**Affiliations:** 1 Department of Emergency Medicine, University of Cincinnati, 231 Albert Sabin Way, ML 0769, Cincinnati, OH USA; 2 Department of Internal Medicine, Department of Health Systems and Implementation Science, Virginia Tech Carilion School of Medicine, Roanoke, VA USA; 3 Medical Education, American Medical Association, 330 N Wabash Ave, Chicago, IL 60611 USA; 4 Department of Medical Education, University of Illinois College of Medicine at Chicago, 1853 W Polk St, Chicago, IL USA; 5 Department of Emergency Medicine, NYU Grossman School of Medicine, New York, NY USA; 6 Department of Pediatrics, University of Washington School of Medicine, Seattle, WA USA; 7 Department of Emergency Medicine, Virginia Commonwealth University, Richmond, VA USA; 8 Veteran Health Administration, Cincinnati, OH USA; 9 Department of Emergency Medicine, Brody School of Medicine at East Carolina University, Greenville, NC USA; 10 Department of Obstetrics and Gynecology, University of Michigan Medical School, Ann Arbor, MI USA

**Keywords:** Health System Science, Professional Development, Evaluation, Curriculum

## Abstract

**Introduction:**

Training in health systems is important for patient care, but often faculty development is lacking. The purpose of the Health Systems Science Scholars Program (HSS-SP) was to teach faculty how to educate learners in Health Systems Science to improve healthcare delivery practices. The objective of this report was to describe program development and evaluate the HSS-SP program in meeting this goal.

**Methods:**

The 12-month HSS-SP focused on curriculum development, assessment design, program evaluation, leadership skills, and scholarship. Training formats included in-person workshops, interactive webinars, community of practice, mentoring, and completion of HSS final projects. This evaluation focused on program completion, satisfaction, learning, and HSS integration in final projects.

**Results:**

One hundred and eight scholars participated across three cohorts. Leaders from 72/155 US medical schools nominated a scholar, indicating support of the program. The retrospective pre-post survey demonstrated significant improvement across all outcome measures including perceived confidence in ability to apply systems thinking, leading change, implementing best practices, commitment to educate others in HSS, improve healthcare delivery, and practice innovative and interactive teaching.

Fifty-six final projects focused on broad implementation of HSS at individual institutions, including 75 specific HSS domains (e.g., social determinants of health and patient safety/quality improvement). Medical school curricula projects arose more often than residency projects (86 versus 22), and most utilized innovative and interactive teaching methods.

**Conclusion:**

The HSS-SP enhanced confidence in and commitment to HSS. Participants’ projects implemented HSS curricular components, furthering dissemination of HSS into the medical education system.

**Supplementary Information:**

The online version contains supplementary material available at https://doi.org/10.1007/s40670-026-02819-z.

## Introduction

The goal of medical education is to train providers to care for patients in complex health systems. Consequently, there is a call to action to produce adaptive and transformative physicians for changing healthcare delivery [1]. To address gaps in health outcomes, medical education is steadily evolving to better prepare providers with competencies in high-value care, quality improvement, population health, informatics, teamwork, and systems thinking to operate across these system issues [1, 2]. Health Systems Science (HSS) addresses these competencies and is defined as the study of how healthcare is delivered, how healthcare professionals work together to deliver that care, and how health systems can improve patient care along with healthcare delivery [2]. HSS as the third pillar of medical education complements the basic and clinical science pillars and integrates the full range of systems-related competencies for the next generation of physicians.

In 2017, Gonzalo et al. proposed HSS Curricula in Undergraduate Medical Education [3]. In addition, in 2020, the International Association of Medical Science Educators (IAMSE) launched a series of webinars to introduce the concepts of HSS to educators “Evolution and Revolution in Medical Education: Health Systems Sciences” [4]. HSS education provides an essential curriculum for physician trainees to learn and practice within complex, demanding healthcare systems [3–8]. Although there are described curricula for teaching individual components of HSS such as quality improvement and social determinants of health, in order for HSS, as a systesm approach, to improve learner education and patient care intentional faculty development and understanding of how to holistically teach HSS is needed [9–12]. No large-scale HSS professional development programs have been described in the literature; however, local programs have been described working with medical school faculty [13–18].

When new conceptual domains such as those in HSS [3] rise in importance for patient care, academic medicine is challenged to develop pathways to meet these new educational requirements for faculty educators. In 2013, to develop those pathways, the American Medical Association’s (AMA) Accelerating Change in Medical Education Consortium initiative funded 11 medical schools to advance medical education in support of adaptive and transformative graduates. The AMA formed an interest group of faculty teaching HSS at their institutions drawn from the Consortium, which crystallized the HSS conceptual model [8, 19]. The model included competencies needed to catalyze health reform (now the Quintuple Aim [20]): improving the patient experience, ensuring the health of populations, reducing the per capita cost of care, sustaining the healthcare team, and addressing health disparities to promote health equity [21].

To intentionally develop faculty and disseminate HSS instruction across medical education, the AMA founded the HSS Scholars Program (HSS-SP) in 2018, to create a national cohort of faculty and administrative leaders to design and teach HSS. The program sought to develop and support faculty across the nation to design, implement, and evaluate HSS curricula across undergraduate (UME) and graduate medical education (GME). The program objectives were to provide faculty implementing and teaching topics related to HSS with the knowledge and skills critical to advancing as teachers, education program administrators, and leaders in HSS. An additional objective was to create a community of practice (COP) to learn from each other and advance the field through curricular implementation development [22, 23]. In this paper, we describe HSS-SP’s development and evaluate outcomes for the first three cohorts (2019–2022). These cohorts represented the completed program data available at the time this evaluation was conducted.

## Methods

### Development of HSS Scholars Program

As part of the initiative, in 2017, the AMA brought together leaders in the field from the Consortium’s HSS faculty development interest group to design the HSS-SP. Following Kern’s Model for Curriculum Design the leadership team conducted a needs assessment to identify faculty development gaps including a survey to the HSS faculty development interest group, a literature review, and pre-survey of a pilot group of scholars(targeted needs assessment). HSS-SP was piloted in 2018 with 25 learners who expressed strong interest in continuing professional development, integrating HSS into undergraduate and graduate medical education, conducting educational research, and adopting systems thinking approaches. These insights informed the design of future and the objectives of the HSS workshops for the cohorts of 2019–2023 for this evaluation [13, 15]. Consequently, working on and providing a report of their HSS teaching/research project was a program requirement, which provided an opportunity for scholars to apply and demonstrate new knowledge and skills.

### Curricular Content

Scholars attended a series of virtual and in-person workshops over 12 months (Online Resource 1). The program adapted to accommodate the COVID pandemic disruptions (Online Resource 2). The HSS-SP started in December with a graduation ceremony of the previous cohort and an introduction for the incoming cohort. During the introductory workshops, scholars received an overview of the HSS conceptual framework from leading experts, including curricular resources. The workshops were created to increase the knowledge, skills, and attitudes of medical education leaders on the design, implementation, and evaluation of HSS curricula at their local institutions. Scholars engaged with presentations of high-quality final projects from the previous cohort of HSS-SP (Online Resource 3). Taken all together, they served to create a COP comprising HSS educators by uniting prior years’ scholars and current scholars.

At the year-long program’s midpoint, scholars then completed a two-day series of workshops. The workshops consisted of essential topics relevant to HSS curriculum development and implementation such as systems thinking, assessment of learning, program evaluation, and change management for curricular integration institutionally; the workshops required active engagement of scholars through pre-assignments and completed work. As a part of these workshops, previous HSS scholars from prior cohorts presented their projects. Scholars attended three AMA-hosted interactive webinars provided by leading experts covering curriculum development, approaches, and challenges to conducting evaluations and guidance on scholarly dissemination of their projects.

### Scholar Projects

Each scholar proposed a project as part of their HSS-SP application. To help ensure success, each scholar was required to submit an institutional letter of support for their projects to document institutional buy-in. They worked on their projects during the year to apply the concepts to curriculum development at their institution. At the end, they submitted a written final report detailing the project objectives, methods, outcomes, degree of implementation, and challenges. (Online Resource 4) It was not expected that all scholars would completely finish their project during the time. Scholars were provided with a certificate of completion, dependent upon attendance and the submission of a final report.

### Community of Practice (COP)

Scholars were grouped into a COP that met periodically throughout the year, consisting of 5–6 scholars with a faculty member and a graduate from a previous cohort [18]. Building on Lave and Wenger’s elements of shared interest, building community, and share practice [22, 23], the COP intended to foster peer learning, feedback, and deeper conceptual understanding through sharing experiences and expertise pertaining to current and developing HSS curricula and leadership issues [22, 23]. Most sessions included review of the members’ projects and sought peer and faculty input for problem solving. These sessions also served to disseminate ideas and address project challenges for the scholars in each group.

### Faculty One-on-One Mentoring

The faculty members met one-on-one with each member of their COP twice a year. These sessions were designed for faculty mentoring of scholars’ projects, providing personalized feedback, and guidance on planning and executing HSS projects. There were often discussions of institutional barriers, curriculum development, learner assessment, and evaluation of the projects.

### Evaluation of the Program

Evaluation of the HSS-SP followed Kirkpatrick and Moore’s program evaluation schema [24, 25]. The Kirkpatrick evaluation model [24] is a common evaluation method reporting reactions, learning, behavior, and outcomes; Moore’s model [25] adds additional context of learners’ participation and potential to impact patient care and community/ population health outcomes. In addition, to understand the program, it was important to know the program’s reach (number of applicants, admitted scholars, and medical schools). Outcome evaluation included (A) the count of scholars who completed the program (participants), (B) retrospective pre- and post-workshop survey responses by scholars focusing on their perceptions, what they felt they had learned, and self- reported behavioral changes; (C) review of the final reports noting HSS domains addressed [3], (D) academic level (UME/GME), (E) type of curricular intervention, and (F) percentage of projects completed.

We analyzed retrospective pre- and post-workshop survey responses using paired t-tests scoring confidence in skills and commitment to HSS teaching after confirming normal distribution. To build content validity evidence, the 11- item survey items focused on the program objectives. For response process validity the survey was designed and piloted iteratively with feedback by the leadership team and intentionally included similar concepts such as teaching and learning in the same stem to decrease the numbers of questions. The responses were from *Not at all = 1; A little = 2; Moderately = 3; A lot = 4.* We calculated Cronbach’s alpha to measure internal consistency and found them to be acceptable (Table 1).

**Table 1:** Pre and post workshop survey results 2019–2022

Questions	Pre (SD)	Post (SD)	$\:\overline{\boldsymbol{d}}$	95% CI	Cohen d	*n*
Please rate your confidence in your skills in each of the following BEFORE/AFTER attending the HSS workshop***						
1. Applying systems thinking principles to your work	2.34* (0.85)	3.24* (0.57)	0.90	[0.75, 1.05]	1.15	106
2. Leading change	2.62* (0.71)	3.12* (0.62)	0.50	[0.36, 0.64]	0.69	106
3. Implementing best practices for teaching and learning	2.50* (0.66)	3.15* (0.62)	0.66	[0.51, 0.80]	0.88	105
4. Integrating HSS into your curriculum	2.86* (0.91)	3.35* (0.61)	0.50	[0.35, 0.64]	0.66	105
5. Evaluating educational programs	2.26* (0.75)	3.04* (0.59)	0.78	[0.69, 0.86]	0.90	104
6. Assessing learners’ HSS knowledge and skills	2.11* (0.78)	3.05* (0.59)	0.93	[0.85, 1.02]	1.06	105
7. Using innovative and interactive teaching strategies	2.64* (0.78)	3.19* (0.54)	0.55	[0.42, 0.68]	0.81	106
Please rate your commitment to each of the following BEFORE/AFTER attending the HSS workshop***						
8. Educating others in the principles of Health Systems Science	3.05* (0.88)	3.68* (0.49)	0.63	[0.53, 0.73]	0.63	103
9. Applying knowledge/skills learned in the Academy to improve care delivery	3.01* (0.78)	3.55* (0.56)	0.54	[0.40, 0.67]	0.77	104
10. Practice various innovative and interactive teaching strategies	2.98* (0.75)	3.49* (0.54)	0.51	[0.44, 0.58]	0.68	104
11. Participate in medical education scholarship in the field of Health Systems Science	2.60* (0.92)	3.44* (0.65)	0.85	[0.67, 1.00]	0.99	105

^*p<.001^

^** Scale: Not at all = 1; A Little = 2; Moderately = 3; A lot = 4^

^***Cronbach’s alpha: pre survey − confidence scale 0.63, commitment scale 0.56; post survey – confidence scale 0.68, commitment scale 0.51^

We reviewed the final reports using a rubric to code the reports of the 108 scholars. Coding rubric noted HSS domain, final form of the project, level of participants, teaching method, stage of project (Table 2). The rubric was derived from an initial review of project reports by RG. Then SAS and RG coded initial reports together for shared mental model and clarification of the rubric, then coded separately and resolved differences by discussion.

**Table 2:** Overall final reports breakdown (*n* = 108*)

Final Report Breakdown	
HSS Domain**	**Number**
• Implementing Curriculum HSS broadly	56
• Population, public, and social determinants of health	39
• Patient safety/quality improvement	39
• Inter-professional care/teaming	24
• Value based healthcare	18
• Healthcare policy and economics	17
• Clinical informatics and health technology	12
• Reports with less than 10 domains (Systems thinking; leadership; change agency, management, and advocacy; professionalism; professional identity; evidence-based practice; ethics and legal)	
Form of Final Project	**Number**
• Curricular Intervention	78
• Needs Assessment Only	15
• Faculty Development	10
• Other (i.e., Research, assessment)	
Academic Level of Participants for Final Projects	**Number**
• UME Curriculum	86
• GME Curriculum	22
Teaching Method**	**Number**
• Problem/Case-based learning	31
• Small group discussion	27
• Didactic lectures/interactive lectures	30
• Modules	25
• Project-based learning/experiential learning	35
• Reflection papers/self assessment of progress	26
• Patient input/interview/simulations/role play	25
• Learner presentations	13
• Team-based learning/problem-based learning	16
• Other (i.e., Discussion papers, Quizzes, Telehealth visit)	
Stage of Project at Submission***	**Number**
• Completed	52
• Implementing	28
• Planning	28

^* 117 scholars accepted, 9 did not finish due to attrition, and 108 graduated^

^** Does not add up to 108 since each final project could use more than one HSS domain and more than one teaching method^

^***Curricular changes vary in terms of how quickly they can be completed. Program completion was based on submission of final report of project^

## Results

### Reach of the Program

From 2019 to 2022, the program received 184 applications, 117 were accepted, and 108 scholars completed the program, (9 did not complete program requirements). The program reached nearly half of operating U.S. medical schools with 72/155(46%) medical schools represented (Online Resource 5). All scholars had teaching and/or leadership responsibilities in their respective academic institutions.

### Survey Responses of Reactions, Learning, and Self-reported Confidence and Commitment

From a retrospective pre/post survey at the end of the course, for each cohort, a total of 11 questions were analyzed to compare changes in confidence and commitment towards HSS education (Table 1). Completion of the survey was not mandatory for completion and response rate varied by survey item. All questions demonstrated asignificant increase in means and a meaningful Cohen’s d from pre-workshop to post-workshop. Participants’ confidence in their ability to– apply systems thinking (Cohen *d* = 1.15), leading change (Cohen *d* = 0.69), and implement best practices for teaching and learning significantly increased (Cohen *d* = 0.88). Their confidence in their ability–integrating HSS into their curriculum (Cohen *d* = 0.66), in evaluating educational programs (Cohen *d* = 0.90), in assessing learners’ HSS knowledge and skills (Cohen *d* = 1.06), and in using innovative and interactive teaching strategies significantly increased (Cohen *d* = 0.81). Their commitment to educating others in the principles of HSS (Cohen *d* = 0.63), to applying the skills they learned in the program for improving healthcare delivery (Cohen *d* = 0.77), to practicing various innovative and interactive teaching strategies significantly increased (Cohen *d* = 0.68). Their commitment to participate in medical education scholarship in the field of HSS also significantly increased (Cohen *d* = 0.99).

### Projects

In the Kirkpatrick evaluation schema, these projects demonstrate organizational results of the program as each scholar implemented HSS at their institution. The organizational impact was inferred due to introducing curricular changes at their home instutions via their final projects, which was further assured due to their home institutions letters of support for their program participation. Through their projects, each graduating scholar developed a new HSS curriculum or enhanced a pre-existing HSS curriculum (Table 2). Of the final projects, the most common topics (*n* = 56) involved implementing a broad HSS curriculum at their institution, followed by education projects focused on population, public, and social determinants of health (*n* = 39), and patient safety/quality improvement (*n* = 39). Seventy-five final projects included two or more HSS domains (e.g., population, public, and social determinants of health, patient safety/quality improvement) [3].

Projects typically centered on UME curricula, such as engaging in curricular reform highlighting HSS, as shown in Table 2. Most projects introduced interactive teaching methods of novel HSS content to develop HSS knowledge and skills of learners through problem- or case-based learning and small group discussion. Most projects focused on curricular interventions for students and residents (*n* = 78), although some projects conducted a needs assessment or focused on faculty development interventions.

HSS curricula across the projects used a variety of education methods. The most common strategies were problem or case-based learning, small group discussions, didactic lectures, and online modules (Table 2). Of the projects, 68 projects mentioned specific evaluation steps taken, with most using subjective surveys for learners. While all scholars submitted project reports to receive certification of completion for the program, some projects were at different phases of development, implemention, and completion.

## Discussion

The HSS-SP aimed to develop faculty to design, implement, and evaluate HSS curricula and thus educate future trainees with the knowledge, skills, and confidence to practice within complex health systems. For the 2019–2022 cohorts, our evaluation explored the program’s outcomes. The program focused on equipping educators with change management skills, systems-thinking frameworks, and interprofessional teaching methods which are key to effective implementation. The program was designed to cultivate a COP among a group of medical educators and healthcare system leaders who will assist in driving the transformation at their institutions to improve healthcare through educating trainees in HSS; although this outcome was not directly studied. The program outcomes documented a significant increase in scholars’ confidence across domains of HSS knowledge and skills as well as increased commitment to HSS curriculum development and scholarship.

Although we do not have specific data, the scholars’ projects, they have the potential to impact the institutions and the broader medical education community through implementation and dissemination of HSS projects at these multiple sites. The implementation of a diverse array of curricular initiatives may influence 72 medical schools. The commitment of participating institutions, through faculty support and commitment of time and resources to implement new HSS curricula, will play a role the success of projects.

The program’s approach, content learning, mentorship, COP, and the development of HSS education projects for the participants’ institutions, offered a comprehensive framework for faculty development that pulled on principles of implementation science and faculty development [26, 27]. The interest in the program, the diversity of the faculty and medical schools represented, as well as the breadth of the projects demonstrates the process of creating a national faculty development program. The combination of webinars, workshops, one-on-one, and group mentoring provided knowledge and skill development for scholars and supported addressing unique institutional challenges and optimizing available opportunities. The COP provided a framework for the exchange of ideas, challenges, and solutions that could be adapted to individual institutions. This collaborative approach contributed to the development of an HSS learning community that connected current scholars with prior scholars, promulgating lessons learned and best practices in HSS education on a national level. The HSS-SP serves as a potential exemplar for creating faculty development on a national level for other critical areas of medical education, such as coaching, equity in assessment, and the utilization of artificial intelligence, among others. Other successful national programs longitudinal faculty development programs include the Harvard Macy Program for Educators in the Health Professions, International Association of Medical Science Educators Medical Educator Fellowship, and others. These serve to create cohorts for learning in specific topic domains.

The HSS-SP successfully graduated multiple cohorts of scholars through 2023. Although the formal program has concluded, its graduates, curricular resources, and national community provide a strong foundation for continued integration of HSS into curricula, assessment, and quality improvement efforts across the medical education continuum. Future work should focus on sustaining and expanding these efforts within medical schools, residency programs, and academic health systems. Additional resources for those developing HSS curricula are listed (Online Resource 6).

This study has some limitations as it represents a cohort of faculty scholars who self-selected into the program and may have already been passionate and dedicated to the HSS framework. Getting buy-in on new curricula from faculty who might not be as dedicated and committed could be more challenging and may not yield similar outcomes. However, this model would still be valid for other faculty development programs where the goal is to empower institutional faculty to lead broader curricular change. The retrospective pre- and post-workshop survey design has inherent limitations of response shift bias, since respondents may not remember how they originally would have responded. While one of the objectives was the development of a community, we did not collect specific measurements of this goal. It would be helpful to collect additional qualitative data about the program and experiences. Finally, while there may be patient-related outcomes, they were not measured in this evaluation.

In summary, HSS-SP trained faculty to design, implement, and evaluate HSS curricula. Our program evaluation suggests that the implementation of a faculty development focused on HSS education has the potential to add value to institutions, their delivery systems, and the larger medical education community by building requisite faculty knowledge, skills, commitment, and creating a network of HSS educators across multiple institutions. Optimizing patient outcomes will require medical educators to incorporate HSS principles throughout the medical education continuum to empower current and future healthcare professionals to transform healthcare delivery.

## Supplementary Information

Below is the link to the electronic supplementary material.

Supplementary Material 1

## Declarations

### Disclaimer

The material in this article was created by the authors and may not reflect the official positions of their respective employers.
