# Supplementary material for: Health Systems Science Scholars Program: National Faculty Development Program
Source: Med Sci Educ. 2026 Jul 13;36(5):2439–46. doi: 10.1007/s40670-026-02819-z (PMC13723530; doi:10.1007/s40670-026-02819-z)
Supplement: Supplementary file 1 [file 40670_2026_2819_MOESM1_ESM.docx]

**Supplementary Information (Online Resources 1-6)**

**Online Resource 1**

**Health Systems Scholars Program**

**Online Resource 2**

**Years in HSS Scholars Program with Content Delivery**

| 2019-2020* | 2020-2021 & 2021-2022 |  |
| --- | --- | --- |
| - October: Webinar #1 Orientation: What is HSS - November: Webinar #2 Planning your project/how to assess if you have institutional support - December: 2-day in person workshop:   - Crosswalk of HSS and systems competencies   - Presentations from 2018 Scholars   - Peer discussion in small groups   - Active teaching methods 1&2   - Assessment   - Group mentoring session 1&2   - Approach to teach QI   - Program evaluation   - Leadership session for local challenges and change management   - Curricular resources   - Action plan development - April: Webinar #3 Thinking about Change with your project - June: Webinar #4 Evaluation Challenges - December: Graduation | - December: Virtual Meeting   - Crosswalk of HSS and systems competencies   - Presentations from 2019 & 2020 Scholars   - Peer discussion in small groups/mentoring session   - Action plan development - February: Webinar #1 Curriculum Development - May: 2-Day virtual workshop   - Systems thinking in medical education   - Informatics, big data, and HSS Education   - High-value care education   - Assessment   - HSS exam preview   - Opportunities and Challenges to teach HSS   - Local challenges and change management   - Open office hours   - Curricular resources and wrap up - June: Webinar #2 Evaluation Challenges - September: Webinar #3 From a Project to Scholarly Work - December: Graduation |  |

^*The program days has met virtually starting in 2020 due to COVID. In 2019, the program varied in form with the start occurring in October with a webinar and another webinar in November followed by the in-person 2-day workshop occurring in December of that year.^

**Online Resource 3**

**Final Project Exemplars***

| **Final Project Titles** | **Description** |
| --- | --- |
| Evaluation of a gamification-style Health System Science Curriculum | The project resulted in 4 gamified Health System Science Modules, which were designed to introduce the “broccoli” of medical education in a palatable way to medical students. 148 students participated in the pilot and evaluation. The project was generally well received by students and students described themselves as more engaged in the meaningful content. |
| Creating a Health Systems Citizen through a GME Distinction Track | The project resulted in a 2 year distinction track within the internal medicine residency (for PGY2-3) at Temple Hospital. The goal was to expose GME house-staff to HSS theory and concepts and long term to develop a cadre leader and change agents, who are also clinician-scholars, that apply systems thinking to accelerate change in medical education and health care delivery. The project found that residents found HSS concepts were important. |
| Building a Collaborative Interdisciplinary Health Systems Science Curriculum across Graduate Medical Education Programs and Systems | The project resulted in a stepwise approach on how to implement a collaborative interdisciplinary curriculum across GME programs along with Psychology graduate programs, which led to a list of lecturers and speakers followed by including interprofessional education (IPE) principles for each lecture. The project acquired 23 GME learners and 10 graduate psychology students who chose to participate in their elective course. |
| We are the Clinical Champions:  An Innovative Approach to Integration of Health Systems Science in the Clinical Years | The project resulted in the recruitment of 9 faculty physicians as HSS Clinical Champions who were trained via year-long faculty development to assist in the deployment of HSS curricular content for clerkship, which was made by HSS Scholar. It was piloted for M3 in Family Medicine (i.e., Value in HC & SOAP-V case conference), OB/GYN (i.e., Health Equity & Structural Racism & Lecture & case-based didactic) and Surgery (i.e., QI/PS and Exploration & presentation ongoing QI initiatives in their dept.). The project found that 88% agreed HSS sessions will “help me approach patient care through a systems-based perspective”. |

^*Presentations of final projects were selected by the director of the HSS Program to showcase quality and range of project topics.^

**Online Resource 4 HSS Final Project Titles**

| **Final Project Titles** | **Author** | **Institution** |
| --- | --- | --- |
| **2019** | | |
| 1.   Implementing a longitudinal, interprofessional Social and Health Systems Science curriculum in the fourth year of medical school | Yee Lam | University of North Carolina School of Medicine |
| 2.   Developing a health systems science toolkit for value-based chronic disease care and education | Valerie Press | University of Chicago |
| 3.   Making HSS Relevant at UF COM through Improving SDOH and Population Health Education | Grant Harrell | University of Florida College of Medicine |
| 4.   The Creation of a Longitudinal, Progressive, 7-Year Health Systems Science Curriculum in a New Medical School | Lisanne C. Hauck | CUNY School of Medicine |
| 5.   Optimization of longitudinal HSS curriculum within varied settings | Amy Christison | University of Illinois College of Medicine at Peoria |
| 6.   Integrating a Health Systems Science Curriculum with a Longitudinal Primary Care Preceptorship | David Gilbert | Brody School of Medicine at East Carolina University |
| 7.   Health Systems Science for a New Curriculum | Jeanne Wilson | New York Medical College |
| 8.   GME Health Systems Science Distinction Track | Rachel Marie E. Salas | Johns Hopkins |
| 9.   Multi-modal Case-Based Patient Safety Education Session for Medical Students | Lori M. Singleton | Morehouse School of Medicine |
| 10.   Evaluating HSS Competencies within Team Training Activities | Susan DeRiemer | Meharry Medical College |
| 11.   Health Policy for the Practicing Physician: A Phase 3 Elective Course | Jordan Warchol | University of Nebraska Medical Center |
| 12.   Clinical Immersion Experience | Paul F. Weber | Rutgers-Robert Wood Johnson Medical School |
| 13.   Creating Health Systems Science Natives starts on Day 1 of Medical School | Stephanie Mann | University of Toledo College of Medicine |
| 14.   Health Systems Science and Interprofessional Education in the VTC School of Medicine | David W. Musick | VTC School of Medicine |
| 15.   Leveraging the Precision Medicine Analytics Platform for Clinical Informatics Education | Ashwini Davison | Johns Hopkins University School of Medicine & Bloomberg School of Public Health |
| 16.   Developing Consensus on Social Determinants of Health Learning Goals Across Four Primary Care Residencies using a Delphi Approach | Iman Hassan | Albert Einstein College of Medicine/Montefiore Medical Center |
| 17.   Propelling Health Systems Sciences to the Forefront Through Undergraduate Medical Education Curriculum Reform | Nadia Bennett | University of Pennsylvania, Perelmand School of Medicine |
| 18.   Development of a Preclinical Medical School Social Justice and Health Equity Curriculum | Megha Garg | University of California, San Francisco (UCSF) School of Medicine |
| 19.   Integrating a Health Systems Science Curriculum for an Internal Medicine Residency Training Program | Lili Shek | Cedars-Sinai Medical Center |
| 20.   Implementation of the Health Systems Science Curriculum in Military Medical Education | Beth Hawks | Uniformed Services University of the Health Sciences (USU) |
| 21.   Case Based Exploration of Health System Science in a Third Year Medical School Course: Science and Art of Medicine Integrated | Anastasia Rowland-Seymour | Case Western Reserve University |
| 22.   Training Mississippi’s future doctors in health systems science: a partnership between the school of medicine and the school of population health at the University of Mississippi Medical Center (UMMC) | Charles Chima | University of Mississippi Medical  Center |
| 23.   HSS in the Clerkship: Application to patient care, guided by Resident near-peer feedback | Monica Yepes-Rios | Case Western Reserve University |
| 24.   UTRGV Health Systems Science GME Grand Rounds | Jennifer Salcedo | University of Texas Rio Grande Valley (UTRGV) School of Medicine |
| 25.   Introduction to Health Systems Science:  Experiential Learning Through Patient  Interviews in the Emergency Department | Nicolas Sawyer | UC Davis School of Medicine |
| 26.   Developing a UME directed HSS curriculum for Stanford Medical School | Ryan Ribeira | Stanford University |
| 27.   Harnessing health systems science to improve continuity in a 3rd year longitudinal integrated clerkship | Meghan Geary | Warren Alpert Medical School of Brown University |
| 28.   Integrating Health Systems Science into the Primary Care Transformation Executive (PCTE) Fellowship at A.T. Still University School of Osteopathic Medicine in Arizona | Ebony Whisenant | A.T. Still University School of Osteopathic Medicine |
| 29.  AMA Health Systems Science Scholars Program | Mitchell J. Gitkind | UMass Medical School |
| 30.   Fundamentals of COVID-19 Course: A Virtual Core Curriculum for Third and Fourth-Year Medical Students | Paul Ko | Indiana University School of Medicine |
| 31.   Project charter completion as a stimulus for quality improvement in the clinical learning environment.  If you build it, they will come. | Karen Segerson | University of Washington School of Medicine |
| 32.   AMA Health Systems Science Scholars Program | Peter Weir | University of Utah |
| 33.   AMA Health Systems Science Scholars Program | Joseph P. Happel | Uniformed Services University |
| 34.   Creating a Longitudinal UME HSS experience | Jay B. Morrow | University of Texas Rio Grande Valley School of Medicine |
| 35.   Highlighting Health Justice in an HSS Curriculum | Ronan Hallowell | University of Southern California |
| 36.   Quality Improvement and Patient Safety | Brian Bales | Vanderbilt University School of Medicine |
| **2020** |  |  |
| 37.   Integrating Health System Science into the Pre-Clerkship Curriculum | Colleen M Croniger | Case Western Reserve University |
| 38.   PSP in Practice: Population Science and Policy in the Clinical Clerkships | Meredith Volle | SIU Medicine |
| 39.   Building a Foundation that Supports the Integration of HSS in Graduate Medical Education | Jonathan A. Snider | HCA Healthcare GME |
| 40.   Expansion and Evaluation of a Health Systems Science and Health Equity Course for Medical Students | Oladimeji Oki | Albert Einstein |
| 41.   Transforming Health Care Systems for Vulnerable and Under-Served Populations | Jane E. Hamilton | McGovern Medical School |
| 42.   The Health & Society Pillar at University of Colorado School of Medicine | Kristin Furfari | University of Colorado School of Medicine |
| 43.   Health Systems Science Innovation Sprints | D. Rani Nandiwada | Upenn School of Medicine |
| 44.   Bridging Health Systems Science with Preparation for Practice:  A Vertically-Integrated, Longitudinal Curriculum in Uncertainty | Dimitrios Papanag0u | Thomas Jefferson University |
| 45.   Implementing a 4-year Health Systems Science thread at University of California Riverside School of Medicine | Remus Popa | University of California Riverside |
| 46.   Creating a Health Systems Citizen through a GME Distinction Track | Dharmini Shah Pandya | Temple University Hospital |
| 47.   Development and Implementation of Health Systems Science Training within University of North Carolina Graduate Medical Education | Casey Olm-Shipman | University of North Carolina |
| 48.   Integrating Health Systems Science Content in the Population Health and Patient, Physician, and Community Courses | Amy Lee | Northeast Ohio Medical University |
| 49.   Building the Infrastructure: Disseminating an Open-access, Modular and Simulated Health Systems Science Curriculum | Andrea Smeraglio | Oregon Health & Science University |
| 50.   A Longitudinal Clerkship Experience In Health Systems Science and Professional Identity Formation | Brian Ford | Uniformed Services University of the Health Sciences |
| 51.   Teaching Health Systems Science in Undergraduate Medical Education Using the Lens of the “Quadruple Aim” | Christine Roth | Baylor College of Medicine |
| 52.   Integrating Artificial Intelligence and Machine Learning into Evidence-Based Medicine Curriculum | Cornelius James | University of South Carolina |
| 53.   Implementation and evaluation of a gamification-style Health System Science Curriculum | Damian Borbolla | University of Miami Hospital |
| 54.   Team-based Learning in Health Systems Science: Developing A Systematic Approach to Curriculum Design and Implementation in Multi-Domain Sessions | Daniel Novak | Keck School of Medicine - University of Souther California |
| 55.   Inventory of health systems science curriculum for the Loma Linda University School of Medicine | Matthew Fong | Loma Linda University |
| 56.   Building a Collaborative Interdisciplinary Health Systems Science Curriculum across Graduate Medical Education Programs and Systems | Tracey Henry | SUNY Upstate Medical University |
| 57.   Interprofessional Experiential Learning Opportunities for Students to Pilot Educational Activities in the HSS Curriculum Revision Process. | Kenneth Snyder | University at Buffalo |
| 58.   Developing a Clinical Informatics Curriculum for Fourth Year Medical Students. | Iris Herrera | Rutgers New Jersey Medical School |
| 59.   HSS Curriculum: Building Upon the Foundation | Joel Reich | Quinnipiac University |
| 60.   Bridging to HSS: Optimization of the QI/PS curriculum for UME Learners on a Virtual Platform | Kanchi Batra | Kaiser Permanente |
| 61.   We are the Clinical Champions: A Dynamic Approach to Integrating Health Systems Science into the Clinical Years” | Natalie E. Karp, | Virgina Tech Carilion School of Medicine |
| 62.   Health Systems Science Clerkship Curriculum | Kim Pfotenhauer | Michigan State University COM |
| 63.   Determining the validity of a low fidelity patient safety simulation exercise to assess medical students’ ability to identify and recommend ways to prevent medical errors | Gursahani, Komo | Washington University |
| 64.   Learning about Public Health through Community Asset Mapping | Laura Hinkle | Indiana University |
| 65.   Don't Blow Your Note Forward and Other EHR Issues: Training Medical Students EHR Stewardship | Lee Revere | McGovern Medical School |
| 66.   Social Determinants of Health Pediatric Education Integration Project | Michael Haight | UC San Francisco-Fresno |
| 67.   A 3-(P)rong Approach to Health Systems Science Training: Programs, Providers and Patients | Nana Coleman | Baylor College of Medicine |
| 68.   Health and Society for the Trek Curriculum | Rita Lee | University of Colorado School of Medicine |
| 69.   A Comprehensive Approach to Chronic Care in the Outpatient Setting: A Quality Improvement Curriculum for The TriStar Southern Hills Family Medicine Residency Program | Ronya Green | TriStar Southern Hills Medical Center |
| 70.   A Step towards Change: Integrating Health Systems Science Curriculum into Fourth Year of Medical School | Sarah Siddiqui | University of Texas Medical Branch |
| 71.   Health System Science: An Experiential and Integrated Approach to Inspire Transformative Leaders | Shirin Shafazand | University of Miami |
| 72.   An Integrated Community-Based Approach to Health Systems Science | Vanessa S. Wong | University of Hawaii |
| **2021** |  |  |
| 73.   Physician’s Role in the Health System | Avtar Nijjer-Sidhu | California Health Science University |
| 74.   Health Systems Science through Service Learning and Community Engagement | Cara Stephenson-Hunter | Albert Einstein College of Medicine |
| 75.   REVAMP: A longitudinal experience in health equity, quality and advocacy | Danny Neghassi | Columbia University Vagelos College of Physicians & Surgeons |
| 76.   Creating Physician Leaders: Work in Progress | Devibala Kelur Govindarajan | Wayne State University School of Medicine |
| 77.   Health System Science Curriculum Development and Implementation: An Inter-professional Collaborative Project in the Medical Sciences Campus of the University of Puerto Rico | Yasmin Pedrogo | University of Puerto Rico |
| 78.   Leadership in Health Systems Science Post-Clerkship Curriculum: Understanding Patient Safety Within Existing Systems | Kasi Chu | Uniformed Services University of the Health Sciences |
| 79.   A Student Co-Created Health Systems Science Systems-Based Learning Modeled Curriculum | Greg Jacobs | Alabama College of Osteopathic Medicine |
| 80.   Imagining and assessing a formal University of Pittsburgh School of Medicine health systems science curricular theme | Andrew Wickerham | University of Pittsburgh School of Medicine |
| 81.   Identifying Community-based Physician Roles | Parvathy Pillai | University of Wisconsin-Madison School of Medicine and Public Health |
| 82.   Using a Problem-Based Learning Case to introduce Social Determinants of Health to First Year Medical Students | Beatriz Tapia | University of Texas Rio Grande School of Medicine |
| 83.   Innovations Health Systems Science Education in the Fourth Year of Medical School | Suzanne Karan | University of Rochester |
| 84.   Health Systems Science (HSS) in the Clerkship Year | Lara Johnson | Texas Tech University Health Sciences Center |
| 85.   Health Systems Science: Content Distribution and Professional Identity Formation | Karin Esposito | Roseman University College of Medicine |
| 86.   A Teaching and Assessment Model for Clerkship Students to improve Clinical Readiness | Johanna Clewing | Texas A&M/Houston Methodist Hospital |
| 87.   Finding best practices in instruction and community care: An interprofessional training approach to health systems science for predoctoral and family medicine residents | Thomas W. Bishop | University of Michigan |
| 88.   Creating an Innovative, Longitudinal Health Systems Science Curriculum at a New Medical School | Courtney Brantley | Wake Forest School of Medicine |
| 89.   Changemakers in Health Systems Science: Careers and Concepts in Practice | Greg Wallingford | University of Austin Dell Medical School |
| 90.   Health Literacy Screening & Intervention by Pre-Clerkship Medical Students | Geoffrey Hayden | Thomas Jefferson University |
| 91.   Developing a health systems science curriculum for learners: integrating high-value care and quality improvement project in an informatics framework. | Sam Pak | University of California San Francisco |
| 92.   Health Systems Curriculum for a Family Medicine Residency Program | Laura Grima | Promedica Monroe Regional Hospital |
| 93.   Exploring and Integrating Health Systems Science Topics into the Core Didactic Activities at the McAllen Family Medicine Residency Program | Heidi Y. Pareja | University of Texas of Rio Grande Valley |
| 94.   Health Systems Science Curriculum for an Internal Medicine Residency Program: Social Determinants of Health with Focus on Health Equity and Racism. | Hoveda Mufti | University of California Riverside School of Medicine |
| 95.   Longitudinal Assessment of Population-Centered Care Domain | Augustine Chavez | Mayo Clinic Alix School of Medicine in Arizona |
| 96.   Integrating health systems science into a medical student curriculum | Maria Robles | Indiana University School of Medicine |
| 97.   Integrating Informatics and Data Science into a New UME Curriculum | Jessica A. Davila | Baylor College of Medicine |
| 98.   Evolving an interprofessional curriculum to incorporate health systems science principles | Jim Carlson | Rosalind Franklin University |
| 99.   Implementing RICE Team: Rapid Improvement in Clinical Excellence | Jonathan Lichkus | Greater Lawrence Family Health Center |
| 100. Health Systems Science Curriculum to Improve Health Care | Janet Lieto | University of North Texas Health Science Center-Texas College of Osteopathic Medicine |
| 101. Radiology-TEACHES (Technology Enhanced Appropriateness Criteria Home for Education Simulation) | Marc Willis | Stanford University |
| 102. Implementing Equity Focused Health Systems Science Learning into Medical Education Using a 0vel Learning Framework | Audra Meadows | University of California San Diego School of Medicine |
| 103.The Effect of Implementation of a Long-Term Clinic Schedule and a Social Determinants of Health Curriculum on Continuity of Care of Newborns and Infants in the Pediatric Resident Clinic-A Quality Improvement Initiative | Premchand Anne | Ascension St. John Children's Hospital |
| 104. Designing a Bi-campus UME “Delivery of Care” Curriculum | David Satin | University of Minnesota |
| 105. HSS GME Champions: An Incubator Program to Integrate Health Systems Science into Graduate Medical Education | Sonia Bharel | Thomas Jefferson University Hospital |
| 106. Contextualizing Health Systems Science for Rural Healthcare Education | Cayla Teal | University of Kansas School of Medicine |
| 107. University of Rochester KEE (K0wledge, Empathy, Equity) Curriculum for Health Systems | Theresa Green | University of Rochester Medical Center |
| 108. Development of a Health Equity and Advocacy Longitudinal Thread Curriculum at the Georgetown University School of Medicine (GUSOM) | Tobie Smith | Georgetown University School of Medicine |

Online Resource 5 Reach of Program

Online Resource 6 Resources (not references)

**Resources for Curriculum Development and Teaching Health Systems Science**

Publications Related to Health Systems Science

1. Leep Hunderfund A.N., et. al, "Finding My Piece in That Puzzle": A Qualitative Study Exploring How Medical Students at Four U.S. Schools Envision Their Future Professional Identity in Relation to Health Systems. *Acad Med*. 2023
2. McDermott C, et. al, Developing a Professional Identity as a Change Agent Early in Medical School: the Students' Voice. *J Gen Intern Med*. 2019
3. Davis C.R., et. al, How Medical Schools Can Promote Community Collaboration Through Health Systems Science Education. *AMA J Ethics*. 2019
4. Walsh D.S., et. al, The Teachers of Quality Academy: Evaluation of the Effectiveness and Impact of a Health Systems Science Training Program. *Am J Med Qual*. 2019
5. Baxley E.G., et. al, The Teachers of Quality Academy: A Learning Community Approach to Preparing Faculty to Teach Health Systems Science. *Acad Med*. 2016
6. Carmody, B.J., et. al, Medical Student Attitudes toward USMLE Step 1 and Health Systems Science - A Multi-Institutional Survey. *Teach Learn Med*. 2021
7. Reilly J.B., et. al, Breaking Down Silos Between Medical Education and Health Systems: Creating an Integrated Multilevel Data Model to Advance the Systems-Based Practice Competency. *Acad Med*. 2023
8. Gonzalo J.D., et. al, A Constructive Reframing of Student Roles and Systems Learning in Medical Education Using a Communities of Practice Lens. *Acad Med*. 2017
9. Gonzalo J.D., et. al, Adding Value to the Health Care System: Identifying Value-Added Systems Roles for Medical Students. *Am J Med Qual*. 2017
10. Gonzalo J.D., et. al, A practical guide for implementing and maintaining value-added clinical systems learning roles for medical students using a diffusion of innovations framework. *Adv Health Sci Educ Theory Pract*. 2018
11. Gonzalo J.D., et. al, Aligning Education With Health Care Transformation: Identifying a Shared Mental Model of "New" Faculty Competencies for Academic Faculty. *Acad Med*. 2018
12. Gonzalo J.D., et. al, Concerns and Responses for Integrating Health Systems Science Into Medical Education. *Acad Med*. 2018
13. Gonzalo J.D., et. al, Curricular Transformation in Health Systems Science: The Need for Global Change. *Acad Med*. 2018
14. Gonzalo J.D., et. al, Educating for the 21st-Century Health Care System: An Interdependent Framework of Basic, Clinical, and Systems Sciences. *Acad Med*. 2017
15. Gonzalo J.D., et. al, Educating patient-centered, systems-aware physicians: a qualitative analysis of medical student perceptions of value-added clinical systems learning roles. *BMC Med Educ*. 2018
16. Gonzalo J.D., et. al, Exploring challenges in implementing a health systems science curriculum: a qualitative analysis of student perceptions. *Med Educ*. 2016
17. Gonzalo J.D., et. al, General Internists as Change Agents: Opportunities and Barriers to Leadership in Health Systems and Medical Education Transformation. *J Gen Intern Med*. 2020
18. Gonzalo J.D., et. al, Health Systems Science Curricula in Undergraduate Medical Education: Identifying and Defining a Potential Curricular Framework. *Acad Med*. 2017
19. Gonzalo J.D., et. al, Health Systems Science in Medical Education: Unifying the Components to Catalyze Transformation. *Acad Med*. 2020
20. Gonzalo J.D., et. al, Health Systems Science: The "Broccoli" of Undergraduate Medical Education. *Acad Med.* 2019
21. Gonzalo J.D., et. al, Implementation and Evaluation of an Interprofessional Health Systems Science Professional Development Program. *Acad Med*. 2023
22. Gonzalo J.D., et. al, New Educator Roles for Health Systems Science: Implications of New Physician Competencies for U.S. Medical School Faculty. *Acad Med*. 2019
23. Gonzalo J.D., et. al, Priority Areas and Potential Solutions for Successful Integration and Sustainment of Health Systems Science in Undergraduate Medical Education. *Acad Med.* 2017
24. Gonzalo J.D., et. al, The triple helix of clinical, research, and education missions in academic health centers: A qualitative study of diverse stakeholder perspectives. *Learn Health Syst*. 2020
25. Gonzalo J.D., et. al, Unpacking Medical Students' Mixed Engagement in Health Systems Science Education. *Teach Learn Med*. 2020
26. Gonzalo J.D., et. al, Value-Added Clinical Systems Learning Roles for Medical Students That Transform Education and Health: A Guide for Building Partnerships Between Medical Schools and Health Systems. *Acad Med*. 2017
27. Borkan J.M., et. al, Health systems science education: The new post-Flexner professionalism for the 21st century. *Med Teach*. 2021
28. Garris J., et. al, Impact of Faculty Training in Health Systems Science on Scholarly Presentation of Resident Physician and Fellow Quality Improvement Projects. *Am J Med Qual*. 2022
29. Andrews J.S., et. al, Expanding innovation from undergraduate to graduate medical education: A path of continuous professional development. *Med Teach*. 2021
30. Lewis, J.H., et. al,  Addressing the Social Determinants of Health in Undergraduate Medical Education Curricula: A Survey Report. *Adv Med Educ Pract*. 2020
31. Kim J., et. al, How a Team Effectiveness Approach to Health Systems Science Can Illuminate Undergraduate Medical Education Outcomes. *Acad Med*. 2023
32. Bartoletta K.M., et. al,  Health Systems Science/Advances in Pediatrics/2021
33. Lomis K., et. al, The Accelerating Change in Medical Education Consortium: Key Drivers of Transformative Change. *Acad Med*. 2021
34. Lawson L., et. al, Developing Tomorrow's Leaders: A Medical Student Distinction Track in Health System Transformation and Leadership. *Acad Med*. 2019
35. Lawson L., et. al, Developing Tomorrow's Leaders: A Medical Student Distinction Track in Health System Transformation and Leadership. *Acad Med*. 2019
36. Dekhtyar M., et. al, Validity of the Health Systems Science Examination: Relationship Between Examinee Performance and Time of Training. *Am J Med Qual*. 2020
37. Long N., et. al, Contributions of Health Professions Students to Health System Needs During the COVID-19 Pandemic: Potential Strategies and Process for U.S. Medical Schools. *Acad Med*. 2020
38. Havyer R.D., et. al,Science of health care delivery milestones for undergraduate medical education. *BMC Med Educ*. 2017
39. Santen S., et. al, Assessing the Transition of Training in Health Systems Science From Undergraduate to Graduate Medical Education. *J Grad Med Educ*. 2021
40. Santen S., et. al, Assessing the Transition of Training in Health Systems Science From Undergraduate to Graduate Medical Education. *J Grad Med Educ*. 2021
41. Mokshagundam S., et. al, Engaging Medical Students in Leadership Development. *Med Sci Educ*. 2019
42. Starr S.R., et. al, Science of Health Care Delivery: An Innovation in Undergraduate Medical Education to Meet Society's Needs. *Mayo Clin Proc Innov Qual Outcomes*. 2017
43. Schneid S.D., et. al, A pipeline for health systems science in postbaccalaureate premedical programmes. *Med Educ*. 2022
44. Lazorick S., et. al, Preparing Faculty to Incorporate Health Systems Science into the Clinical Learning Environment: Factors Associated with Sustained Outcomes. *Am J Med Qual*. 2022
45. Elliot, V., et. al, Triple Challenge: How Medical Students May Solve 3 Long-Standing Problems Bedeviling Health Systems and Medical Education. *Acad Med*. 2021

**Health System Science Books**

- Skochelak SE, ed. *Health Systems Science*. Second edition. Elsevier; 2021.
- Ehrenfeld J, ed. *Health Systems Science Review*. First Edition. Elsevier; 2019.
- Maben-Feaster R, ed. *Health Systems Science Education: Development and Implementation*. First Edition. Elsevier; 2022.
- Gonzalo, J, ed. *Value-Added Roles for Medical Students*, Elsevier; 2022

**AMA HSS Modules can be found here:** [**https://edhub.ama-assn.org/health-systems-science**](https://edhub.ama-assn.org/health-systems-science)

o   What Is Health Systems Science?

o   Introducing Health Care Policy and Economics

o   How Clinical Informatics Impacts Health Care Delivery

o   What Are the Components of Value-Based Care?

o   Establishing Essential Leadership Behaviors

o   What Makes Team-Based Care Effective?

o   Recognizing A Physician's Role in Patient Safety

o   What Are Social Determinants of Health?

o   Understanding and Improving Population Health

o   Essentials of Quality Improvement

o   What to Know About Health Care Delivery Systems

o   How Systems Thinking Applies to Health Care
